# Malaria vector bionomics and transmission in irrigated and non-irrigated sites in western Kenya

**DOI:** 10.1007/s00436-022-07678-2

**Published:** 2022-10-07

**Authors:** Benyl M. Ondeto, Xiaoming Wang, Harrysone Atieli, Pauline Winnie Orondo, Kevin O. Ochwedo, Collince J. Omondi, Wilfred O. Otambo, Daibin Zhong, Guofa Zhou, Ming-Chieh Lee, Simon M. Muriu, David O. Odongo, Horace Ochanda, James Kazura, Andrew K. Githeko, Guiyun Yan

**Affiliations:** 1 Department of Biology, University of Nairobi, Nairobi, 00100 Kenya; 2 Sub-Saharan Africa International Center of Excellence for Malaria Research, Tom Mboya University, Homa Bay, 40300 Kenya; 3 Program in Public Health, College of Health Sciences, University of California at Irvine, Irvine, CA 92697 USA; 4 Department of Biochemistry, Jomo Kenyatta University of Agriculture and Technology, Nairobi, 00200 Kenya; 5 Department of Zoology, Maseno University, Maseno, Kenya; 6 Department of Biological Sciences, Pwani University, Kilifi, 80108 Kenya; 7 Center for Global Health and Disease, Case Western Reserve University, Cleveland, OH 44106 USA; 8 Centre for Global Health Research, Kenya Medical Research Institute, Kisumu, 40100 Kenya

**Keywords:** Irrigation, Vector density, Vector bionomics, Malaria transmission, *Anopheles*

## Abstract

Irrigation not only helps to improve food security but also creates numerous water bodies for mosquito production. This study assessed the effect of irrigation on malaria vector bionomics and transmission in a semi-arid site with ongoing malaria vector control program. The effectiveness of CDC light traps in the surveillance of malaria vectors was also evaluated relative to the human landing catches (HLCs) method. Adult mosquitoes were sampled in two study sites representing irrigated and non-irrigated agroecosystems in western Kenya using a variety of trapping methods. The mosquito samples were identified to species and assayed for host blood meal source and *Plasmodium *spp. sporozoite infection using polymerase chain reaction. *Anopheles arabiensis* was the dominant malaria vector in the two study sites and occurred in significantly higher densities in irrigated study site compared to the non-irrigated study site. The difference in indoor resting density of *An*. *arabiensis* during the dry and wet seasons was not significant. Other species, including *An*. *funestus*, *An*. *coustani*, and *An*. *pharoensis*, were collected. The *An*. *funestus* indoor resting density was 0.23 in irrigated study site while almost none of this species was collected in the non-irrigated study site. The human blood index (HBI) for *An*. *arabiensis* in the irrigated study site was 3.44% and significantly higher than 0.00% for the non-irrigated study site. In the irrigated study site, the HBI of *An*. *arabiensi*s was 3.90% and 5.20% indoor and outdoor, respectively. The HBI of *An*. *funestus* was 49.43% and significantly higher compared to 3.44% for *An*. *arabiensis* in the irrigated study site. The annual entomologic inoculation rate for *An*. *arabiensis* in the irrigated study site was 0.41 and 0.30 infective bites/person/year indoor and outdoor, respectively, whereas no transmission was observed in the non-irrigated study site. The CDC light trap performed consistently with HLC in terms of vector density. These findings demonstrate that irrigated agriculture may increase the risk of malaria transmission in irrigated areas compared to the non-irrigated areas and highlight the need to complement the existing malaria vector interventions with novel tools targeting the larvae and both indoor and outdoor biting vector populations.

**Graphical abstract:**

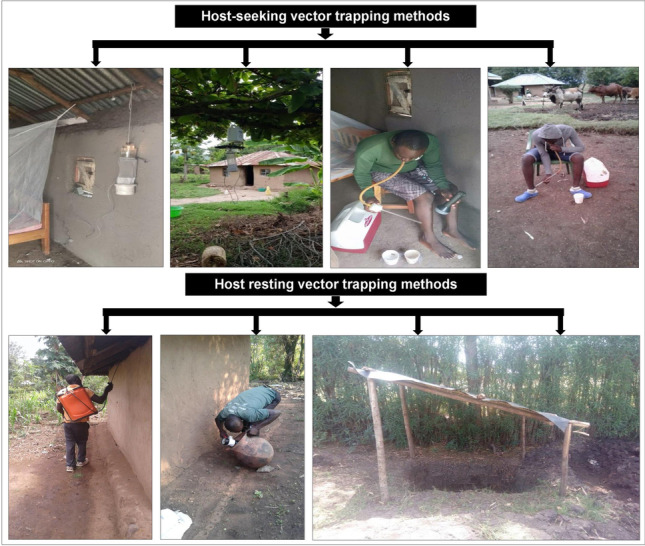

**Supplementary Information:**

The online version contains supplementary material available at 10.1007/s00436-022-07678-2.

## Introduction

In Africa, food insecurity and famine continue to affect millions of people (Baro and Deubel 2006). Given that nearly half of potential arable land in Africa occur in areas with irregular rainfall pattern, many countries have adopted irrigated agriculture as a key strategy to meet the rising demand for food (Blank et al. 2002). This effort has improved crop production by enabling the reclamation of arid and semi-arid lands, enhancing crop yield, extending the crop-growing season, and reducing the risk of crop failure (Oomen et al. 1988; Keiser et al. 2005; Yohannes et al. 2005). In addition, irrigation projects have led to improved nutrition and socioeconomic conditions for the vulnerable population (Bryan et al. 2019). Despite these socioeconomic benefits, irrigated agriculture creates numerous water bodies that may support large populations of mosquitoes including malaria vectors although this may not necessarily lead to increased risk of malaria transmission (Patz et al. 2004; Muturi et al. 2008a).

In Sudan, introduction of the Gezira-Managil scheme in the Nile River Valley led to an increased densities of *An*. *arabiensis* exacerbating malaria outbreaks (Oomen et al. 1988). Similarly, irrigation schemes increased vector densities and malaria incidences in South Central Sierra Leone (Gbakima 1994), Ethiopia (Yohannes et al. 2005), Cameroon (Robert et al. 1992), and Burundi (Coosemans 1985). In contrast, reduction in malaria transmission was reported in irrigated rice cultivations of Mali (Sissoko et al. 2004) and Lower Moshi Tanzania (Ijumba et al. 2002a) as compared to the adjacent non-irrigated areas. Reduced transmission could be attributed to increased wealth that was implicated in the increased acquisition and use of insecticide treated nets and anti-malarial drugs in irrigation projects leading to reduced malaria incidence (Ijumba et al. 2002a; Henry et al. 2003; Diuk-Wasser et al. 2005). However, in some cases, introduction of irrigation schemes like in Senegal River Delta had no impact on malaria transmission (Faye et al. 1995). Worthwhile noting is that in areas of stable malaria transmission, the introduction of irrigated agriculture has little or no impact on malaria transmission (Ijumba and Lindsay 2001; Ijumba et al. 2002b) nevertheless in semi-arid savannah zone of Africa irrigated rice cultivation can alter malaria transmission pattern from seasonal to perennial (Dolo et al. 2004; Sissoko et al. 2004). Hence, the impact of water development projects on malaria transmission is variable and likely depends on the ecology of local mosquito vectors, underlying ecological factors, epidemiologic setting, socioeconomic conditions, and existing malaria control measures (Keiser et al. 2005). Thus, its complexity can only be understood through site-specific evaluation of these parameters.

Insecticide-based vector control interventions mainly long-lasting insecticidal nets (LLINs) and indoor residual spraying (IRS) have been implemented to reduce malaria transmission with significant impacts. These tools have resulted in dramatic reduction in the proportion of endophagic and anthropophilic malaria vector species such as *Anopheles gambiae*, *An*. *coluzzii*, and *An*. *funestus* and a proportionate increase in *An. arabiensis*, which tend to be exophagic and less anthropophilic. However, previous studies indicate that vectors can develop resistance to insecticides or adapt to the presence of insecticides by becoming partially zoophilic and exophilic. Hence with the scale up of LLINs and widespread use of IRS, there is likely to be a shift in vector dominance from the highly endophilic *An*. *gambiae*/*An*. *coluzzi* and *An*. *funestus* to the more zoophilic and exophilic *An*. *arabiensis* (Bayoh et al. 2010; Futami et al. 2014; Abong’o et al. 2020).

There is a pressing need to enhance our understanding on the effect of irrigation in a site where there is malaria vector control. The study aims to assess the effect of a recently established irrigation scheme in Homa Bay, Kenya, on malaria vector bionomics and transmission. Vector control intervention using LLINs and IRS with organophosphate, pirimiphos-methyl (Actellic® 300CS) was being undertaken during the study period. Long-term success of the current malaria control efforts, ITNs and IRS, is dependent on continuous operational surveillance of the mosquito vectors, thus an effective mosquito sampling tool is required. Hence, the secondary goal was to compare the trap effectiveness of Centers for Disease Control and Prevention (CDC) light traps against the gold standard, human landing catches (HLCs). Results of this study will serve as the baseline vector bionomics and malaria transmission pattern for the evaluation of the success of core vector interventions and inform policymakers in planning and guiding future interventions especially in irrigated areas where there is scale up of LLINs distribution and application of IRS.

## Material and methods

### Study site

**Fig. 1:**
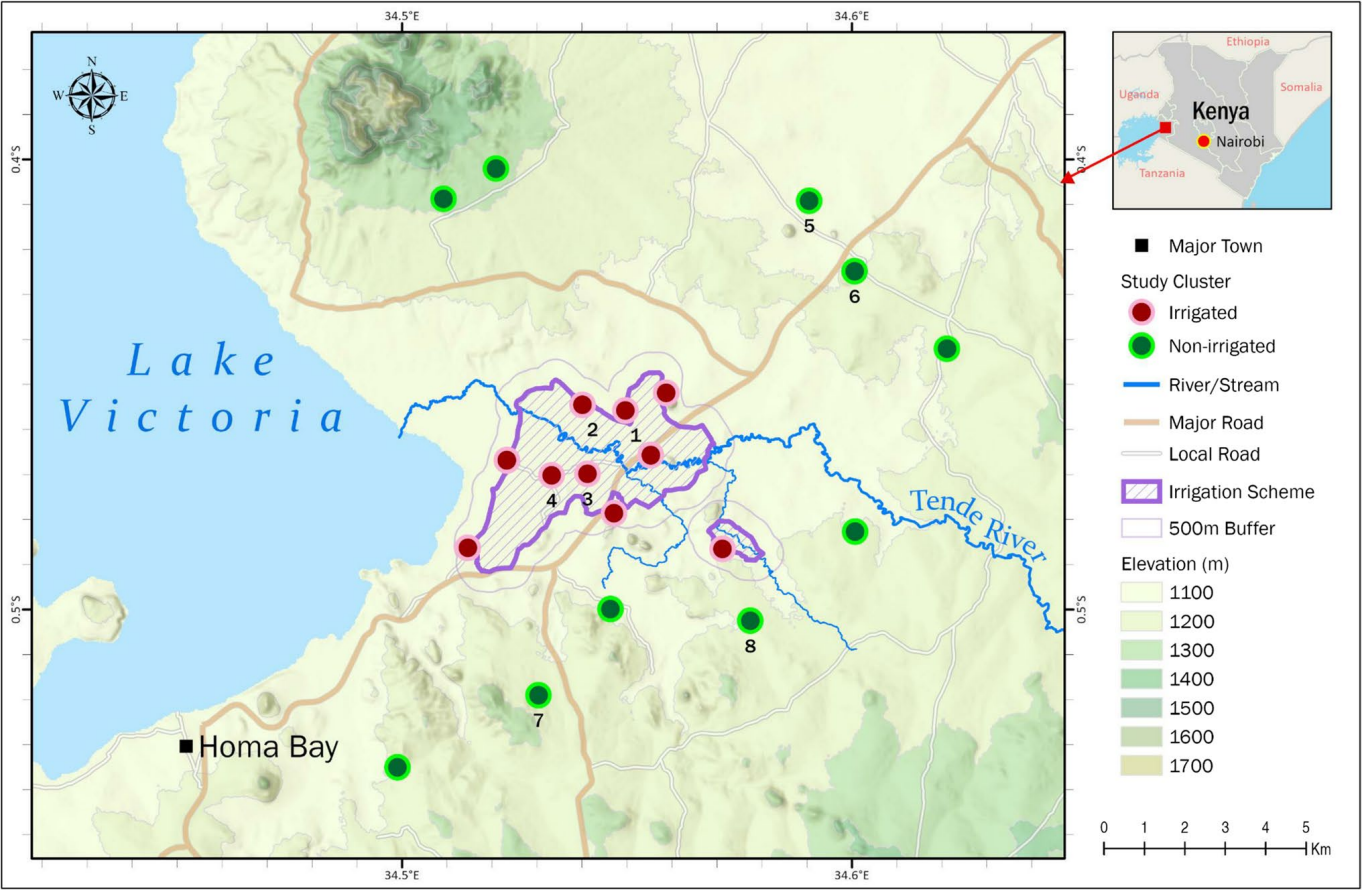
The site map indicates the study clusters in Homa Bay, Kenya. The red dots represent the clusters within the irrigated zone and the green dots represent the clusters within the non-irrigated zone. Clusters labeled with numbers 1–8 also have been surveyed monthly for malaria vectors population dynamics research

The study was conducted in Rangwe (0°35′24″S; 34°35′05″E) and Rachuonyo South (0°19′17″S; 34°07′22″E) sub-counties in Homa Bay County of western Kenya situated at an altitude of 1,202 m above sea level adjacent to the eastern shore of Lake Victoria (Fig. 1). The county is a semi-arid expansive lowland characterized by black cotton soils. The area experiences bimodal rainfall pattern with a mean annual of 1,226 mm. The long rainy season occur between April and June while short rainy season occur from October to November. The hot and dry season is from January to March. The mean annual temperature is 25.7 °C, with a minimum of 18.3 °C and maximum of 29 °C. Relative humidity varies from 52 to 67%. The main economic activities are fishing in Lake Victoria and irrigated and non-irrigated subsistence farming.

The study was conducted in Kimira-Oluch Irrigation Scheme (0°26′44″S; 34°31′28.0″E) and its vicinity, which lies in an area of 110 km^2^ and is located approximately 10 km north of the town of Homa Bay, Homa Bay County of western Kenya. The study site was stratified into irrigated and non-irrigated zones depending on proximity to irrigation scheme. Each zone consisted of 10 clusters (cluster radii vary from 0.25 to 1 km) and with populations ranging from 50 to 250 residents in each cluster. The irrigated zone is within a concrete canal and flood irrigation systems. The crops grown under this irrigation scheme mainly include maize, beans, kales, tomatoes, pawpaw, bananas, watermelons, and rice grown in paddies. The non-irrigated zone is located about 5–10 km from the irrigated zone.

In an effort to reduce malaria burden in the lake endemic zone, vector control interventions were instigated between 2006 and 2008 through the use of LLINs and IRS. The first mass LLIN distribution occurred in 2006 followed by successive rounds of distribution in 2011, 2014, 2017, and 2021 (Ministry of Health 2016; 2021a; Ng’ang’a et al. 2021). Insecticide residual spraying was first implemented in Rachuonyo district in 2008 followed by successive rounds in 2009 to 2012 and 2017 to 2021 in targeted areas (PMI 2013; Gimnig et al. 2016; 2021b). According to a recent study conducted by Orondo et al. (2021) in the study site, the use of LLINs and IRS in the irrigated and non-irrigated zones is similar.

### Study design

**Fig. 2:**
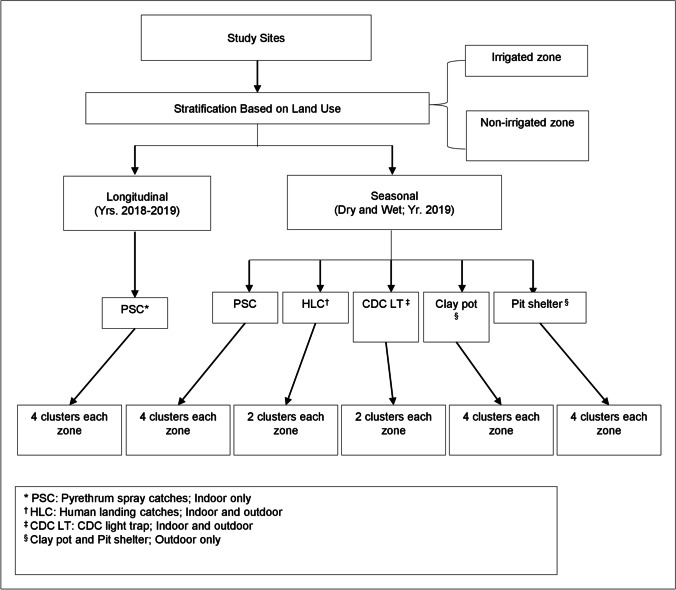
Study design flow chart

Seasonal surveys were conducted in the dry (Jan–Mar) and wet (Apr–Jun) seasons in 2019 using five different trapping methods (Fig. 2). Indoor and outdoor host-seeking vector collections using CDC light traps and HLCs were undertaken in two randomly selected clusters in each zone. There were 160 trap-nights for each trap. Indoor and outdoor resting vector collections using pyrethrum spray catches (PSCs) (indoor), clay pots (outdoor), and pit shelters (outdoor) were undertaken in four randomly selected clusters in each zone. There was a total of 320 trap-nights for each trap and 144 for pit shelters. Longitudinal adult vector surveillance was conducted using PSCs in four clusters in each zone for malaria vectors population dynamic research between 2018 and 2019 (Fig. 2).

### Seasonal survey

#### CDC light traps

**Fig. 3:**
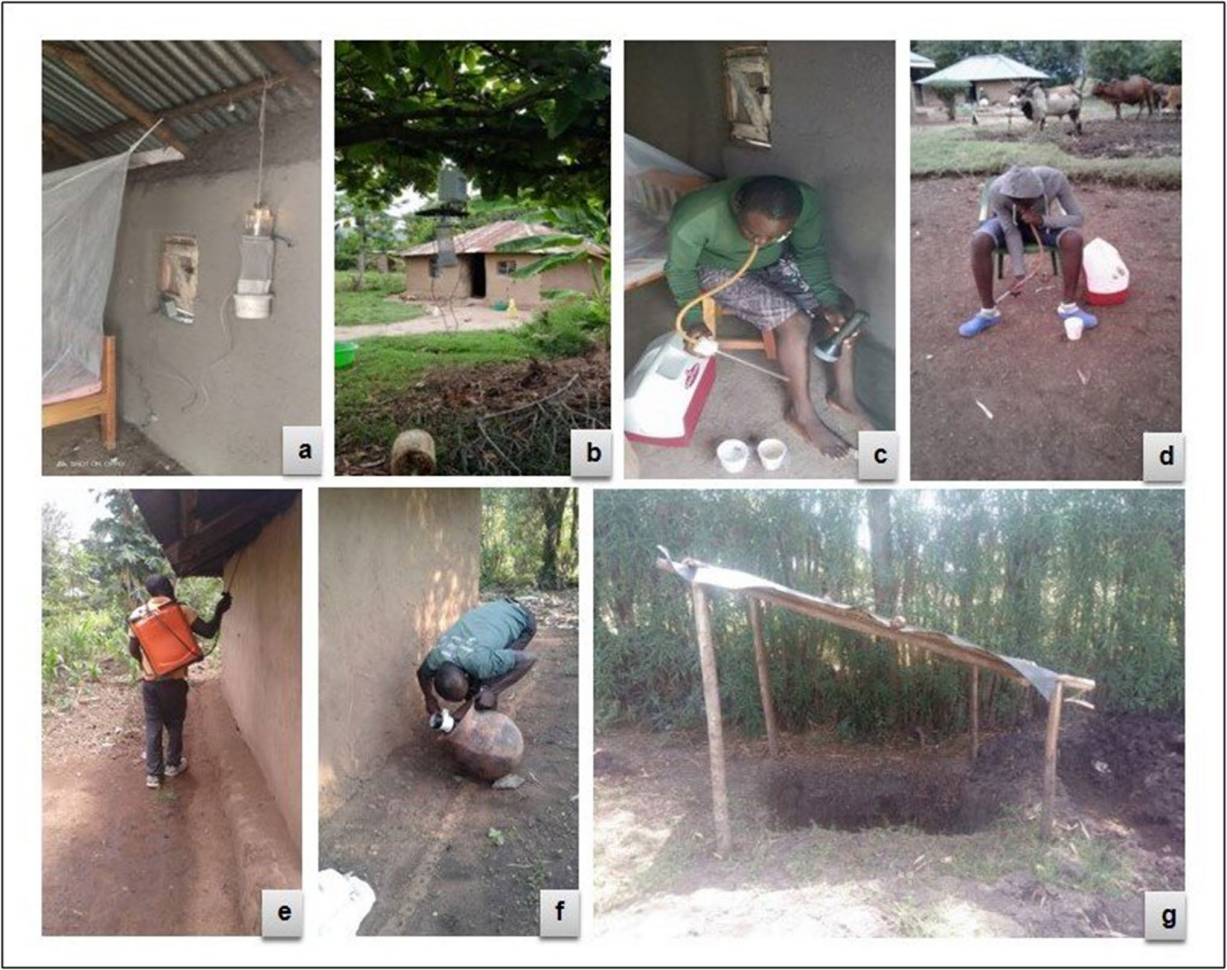
Vector sampling tools [**a** indoor CDC light trap, **b** outdoor CDC light trap, **c** indoor human landing catches, **d** outdoor human landing catches, **e** pyrethrum spray catches, **f** clay pot, **g** pit shelter] used for outdoor and/or indoor host-seeking/resting malaria vector (pictures captured in the field)

The CDC light traps were set both indoor and outdoor to assess vector host-seeking behavior (Fig. 3a, b) (WHO 1975). The indoor CDC light trap was set 1 m beside an occupied bed at a height of 1.5 m off-ground and the outdoor trap was set within 5 m away from the front door at a height of 1.5 m off-ground. Vector collections were undertaken in five randomly selected houses in each cluster from 6 p.m. to 6 a.m. for four consecutive nights once per season.

#### Human landing catches

Human landing catches were conducted both indoor and outdoor to assess vector host-seeking behavior (Gimnig et al. 2013; WHO 2013). In each compound, vectors were collected indoors (at the house entrance) and outdoors 5 m away from the sentinel indoor collection house (Fig. 3c, d). Collection was undertaken by four volunteers, two in each of the indoor and outdoor stations, who alternated after 6 h. Hourly collections were done from 6 p.m. to 6 a.m. each night, with 45 min of collection and a 15 min break per hour. Each hourly collection was placed in individually labeled paper cups and maintained with a 10% sugar solution pad and then placed in a cool box. The same collectors conducted HLCs every night and were rotated between positions (indoor vs. outdoor). All collections were supervised by a team leader. Vector collections were undertaken in five randomly selected houses in each cluster for four consecutive nights once per season. All collectors were provided with anti-malarial chemo-prophylaxis during the study period.

#### Pyrethrum spray catches

Indoor resting vector collections using PSCs (Fig. 3e) were undertaken in 20 randomly selected houses in each cluster once per season from 6 a.m. to 9 a.m. following WHO protocol (WHO 1975).

#### Clay pots

Outdoor resting vectors were assessed using clay pot outdoors (≤ 5 m away from the house) placed behind the house close to the bedroom where there is minimal human activities to minimize disturbance (Fig. 3f). The 20 l capacity clay pots were ∼0.5 m in height, 45 cm in diameter on wide base, and a 20 cm diameter opening as described by Odiere et al. with modifications (Odiere et al. 2007). During setting, the pots were filled with 2 l of rainwater to increase humidity (Ng’Habi et al. 2010) and tilted at 45° to the ground. Vector collections were undertaken in 20 randomly selected houses in each cluster once per season. One pot was set at 6 p.m. in each of the 20 houses and mosquito collected the following morning between 6 a.m. and 9 a.m. using a hand-held Prokopack aspirator.

#### Pit shelters

Pit shelters were dug (1.5 m in depth, 1.5 m in length, and 1 m in width) within 20 m of each selected house according to the method of Muirhead-Thomas (Fig. 3g) (Muirhead-Thomson 1958). In each of the four vertical sides, approximately 0.6 m from the bottom of the pit, cavities were dug to a depth of about 0.3 m. The mouth of the main pit was shaded from above using an artificial shelter. Vector collections were undertaken in one randomly selected house in each cluster for five consecutive nights monthly per season. Vector collection was undertaken between 6 a.m. and 9 a.m. inside the cavities by using a hand-held Prokopack aspirator according to WHO protocol (WHO 1975).

### Longitudinal surveillance

Temporal indoor resting vector population abundance was determined by conducting monthly surveys by PSCs in five randomly selected houses in each cluster. Application of IRS was undertaken in the study area by the National Malaria Control Program (Kenya) during the dry seasons in February of 2018 and 2019.

### Vector species identification

All adult mosquitoes collected were transferred to the International Center of Excellence for Malaria Research (ICEMR) laboratory in Homa Bay, sorted, and anophelines identified morphologically to species as previously described (Gillies and Coetzee 1987). Female *Anopheles* mosquitoes were physiologically classified according to their gonotrophic stages: unfed, blood-fed, half-gravid, and gravid. For species identification, DNA was extracted from the legs and wings of each specimen using the Chelex protocol by Musapa et al. (2013). Sibling species in *An*. *gambiae* s.l. and *An*. *funestus* were speciated by conventional polymerase chain reaction (PCR) as described by Scott et al. (1993) and Koekemoer et al. (2002), respectively.

### Molecular detection of blood meal sources and sporozoite infections

The abdomen of *Anopheles* mosquito specimens was carefully separated from the head and thorax and DNA extracted (Musapa et al. 2013). The blood meal sources of each freshly fed *Anopheles* mosquitoes were analyzed by multiplexed PCR as described by Kent and Norris (2005).

The DNA extracted (Musapa et al. 2013) from the head and thorax of each mosquito specimen was used to determine sporozoite infections of *Plasmodium* spp. by using the multiplexed real-time quantitative PCR (qPCR) assay. The assay was performed using the published species-specific 18 s ribosomal RNA probes and primers for *Plasmodium falciparum*, *P. malariae*, and *P. ovale* (Shokoples et al. 2009; Veron et al. 2009).

### Data management and analysis

Data were entered in Microsoft Excel 2010 datasheets and analyses were done using R statistical software (version 4.0.3; R foundation for statistical computing, Vienna, Austria). Mean density (95% confidence interval, CI) and proportions were calculated for vector populations. The density of adult anopheline mosquitoes was calculated as the average number of female mosquitoes per house per night (f/h/n). Several models were evaluated for the analysis of vector density, and the model with the lowest akaike information criterion (AIC) and variables of interest was selected as the best model (Additional file 1). In the analysis of seasonal data, a negative binomial mixed model (NBMM) was fitted to analyze *Anopheles* densities outdoor, indoor, and trapping method (HLC and CDC light trap) (Additional file 1; Table S1, Table S2, Table S3). Zone and trapping methods were fitted as the fixed variables in the outdoor and indoor models while zone, trapping methods, and location (indoor and outdoor) were considered as fixed variables in the trapping method model. In the indoor and trapping method models, house number and date were used as covariates whereas house number and cluster were used as covariates in the outdoor model. In the analysis of longitudinal data, a NBMM with repeated measures were fitted to compare *Anopheles* densities and seasonality in the two zones by adjusting for months (Additional file 1; Table S4). Zone and season were fitted as the fixed variables and year: (date: cluster), date: cluster, cluster considered as covariates. The chi-square test was used to compare differences in vector species gonotrophic stage proportions between indoor and outdoor collections and also the zones. The human blood index (HBI) for each mosquito species was calculated as the proportion of mosquito samples that had fed on humans out of the total number tested (Garrett-Jones 1964). Sporozoite rates were calculated as the proportion of *Anopheles* mosquito samples positive for *Plasmodium* spp. out of the total number tested. The human biting rate was calculated as the product of blood-fed females per person per night and the human blood index. Annual entomological inoculation rates (EIRs) were calculated as the product of the sporozoite rate and the human biting rates (Macdonald 1957).

## Results

### Seasonal survey

#### Vector species composition

A total of 3,556 female *Anopheles* mosquitoes belonging to four species were collected using the five trapping methods during the study period. *Anopheles gambiae* s.l. was the predominant anopheline species accounting for 79.2%, followed by *An*. *coustani* (15.6%), *An*. *pharoensis* (4.6%), and *An*. *funestus* group (0.6%). In addition, 1,140 male *Anopheles* mosquitoes and 17,387 *Culex* species were collected (males, *n* = 3,776; females, *n* = 13,611). A total of 958 specimens (941 *An*. *gambiae* s.l. and 17 *An*. *funestus*) were analyzed for sibling species identification. Of these, 765 (81.3%) *An*. *gambiae* s.l. and 7 (41.2%) *An*. *funestus* were successfully amplified and all were confirmed as *An*. *arabiensis* and *An*. *funestus* s.s., respectively.

#### Indoor and outdoor vector density

**Fig. 4:**
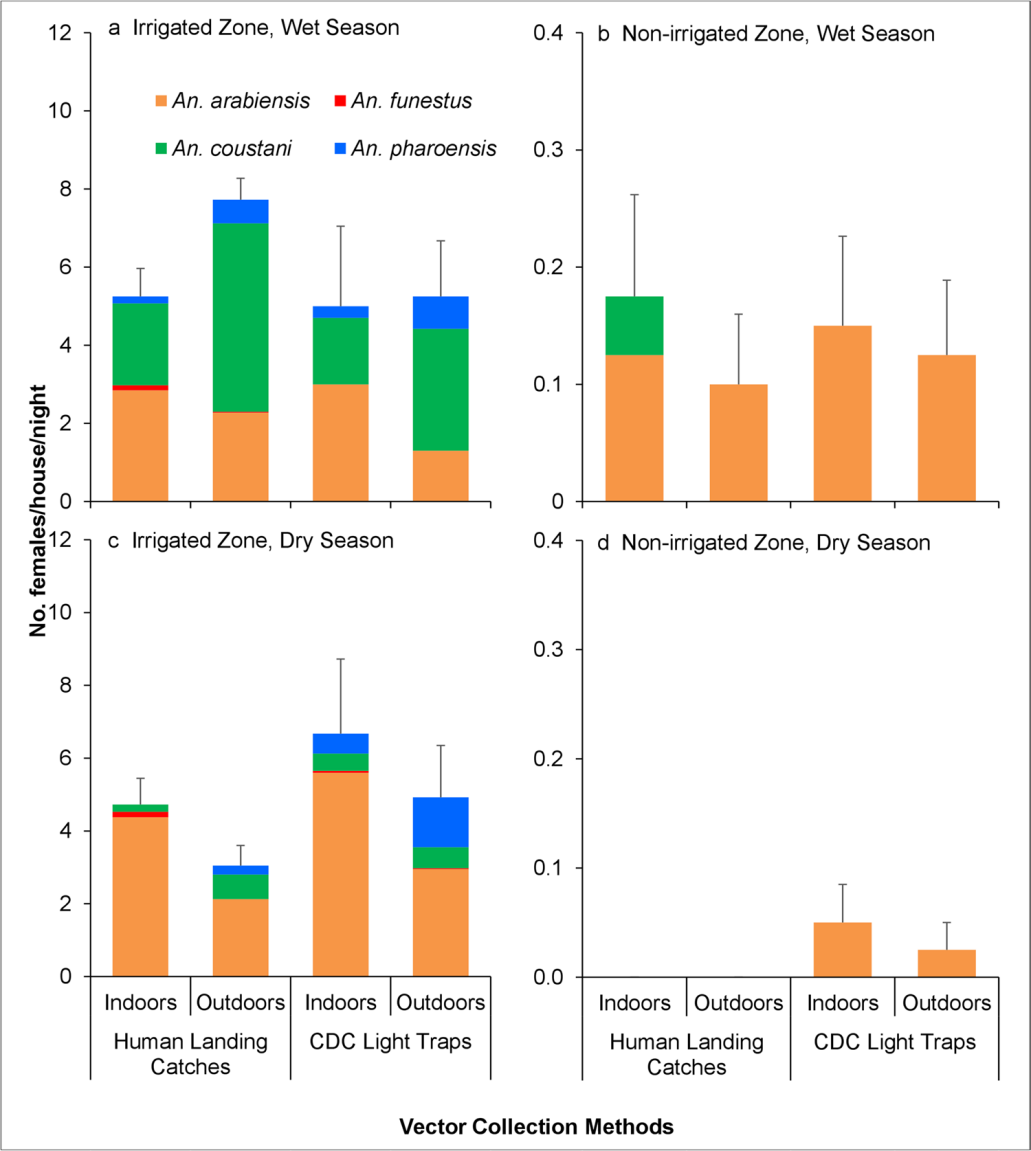
Host-seeking female *Anopheles* mosquito densities collected using human landing catches and CDC light traps indoors and outdoors from irrigated and non-irrigated zones in dry (Jan–Mar) and wet (Apr–Jun) seasons in 2019. Error bars were for the standard error for the total *Anopheles* mosquitoes collected. (*n* = 160 trap-nights for each trap)

The mean density of the female *An*. *arabiensis* mosquitoes varied by zone and collection method (Table 1, Fig. 4, and Additional file 1). Only few *An*. *funestus* were collected in the trapping methods, and the mean density was not analyzed.

**Table 1:** Densities of resting female *Anopheles* mosquito collected using pyrethrum spray catches (PSCs) (indoor), clay pot (outdoor), and pit shelter (outdoor) from irrigated and non-irrigated zones pooled of dry (Jan–Mar, 2019) and wet (Apr–Jun, 2019) seasons (*n* = 320 trap-nights for each trap; *n* = 144 trap-nights for pit shelter) [mean (95% CI)]

Study site and species	Dry season			Wet season		
	Indoor	Outdoor		Indoor	Outdoor	
	PSC	Clay pot	Pit shelter	PSC	Clay pot	Pit shelter
Irrigated zone						
*An*. *arabiensis*	4.36	3.34	9.75	2.08	1.88	11.42
	(2.89, 5.84)	(2.29, 4.39)	(5.26, 14.24)	(0.88, 3.27)	(1.27, 2.48)	(8.50, 14.34)
*An*. *funestus*	0.01	0.03	0	0	0	0.04
	(0, 0.04)	(0, 0.06)				(0, 0.09)
*An*. *coustani*	0	0	0.15	0.01	0.01	0.04
			(0, 0.32)	(0, 0.04)	(0, 0.04)	(0, 0.09)
Non-irrigated zone						
*An*. *arabiensis*	0.06	0.11	0.30	0.35	0.21	0.77
	(0.01, 0.12)	(0.02, 0.21)	(0, 0.73)	(0.15, 0.55)	(0.09, 0.33)	(0.48, 1.05)
*An*. *funestus*	0	0	0	0	0	0.04
						(0, 0.09)

In the irrigated zone, the outdoor mean density of *An*. *arabiensis* was significantly higher compared to the non-irrigated zone (*Z* =  − 8.276, df = 776, *P* < 0.001) (Table 1, Fig. 4, Additional file 1: Table S1). Similarly, in the irrigated zone, the indoor mean density of *An*. *arabiensis* was significantly higher compared to the non-irrigated zone (*Z* =  − 9.403, df = 628, *P* < 0.001) (Table 1, Fig. 4, Additional file 1: Table S2).

Pit shelters (*Z* = 6.433, df = 776, *P* < 0.001) and clay pots (*Z* = 3.117, df = 776, *P* < 0.01) yielded a significantly higher outdoor density of *An*. *arabiensis* than CDC light traps, whereas the difference in outdoor mean density of *An*. *arabiensis* between HLC and CDC light trap was not significant (*Z* = 0.966, df = 776, *P* = 0.334) (Additional file 1: Table S1). There was no significant difference in the indoor mean density of *An*. *arabiensis* from PSC and HLC compared to CDC light traps (all, *P* > 0.001) (Additional file 1: Table S2).

Overall, the mean density of *An*. *arabiensis* was higher in the irrigated zone than in the non-irrigated zone. There was no significant difference in the mean density of *An*. *arabiensis* during the dry and wet seasons (all, *P* > 0.001) (Additional file 1: Table S1, Table S2, and Table S3).

#### HLC and CDC light trap comparison

The HLC and CDC light trap yielded a significantly higher host-seeking density of *An*. *arabiensis* in the irrigated zone than non-irrigated zone (*Z* =  − 9.841, df = 631, *P* < 0.001) (Fig. 4, Additional file 1: Table S3). There was no significant difference between HLC and CDC light traps in terms of the *An*. *arabiensis* mean host-seeking density (*Z* = 0.351, df = 631, *P* = 0.725) (Fig. 4, Additional file 1: Table S3). The results indicated that CDC light trap performed consistently with HLC in terms of vector density. The mean indoor and outdoor host-seeking density of *An*. *arabiensis* from HLC and CDC light traps collections varied significantly (*Z* =  − 3.175, df = 631, *P* < 0.01) with the highest mean host-seeking density collected indoors (Fig. 4, Additional file 1: Table S3).

#### Gonotrophic status of female *Anopheles* mosquitoes

**Fig. 5:**
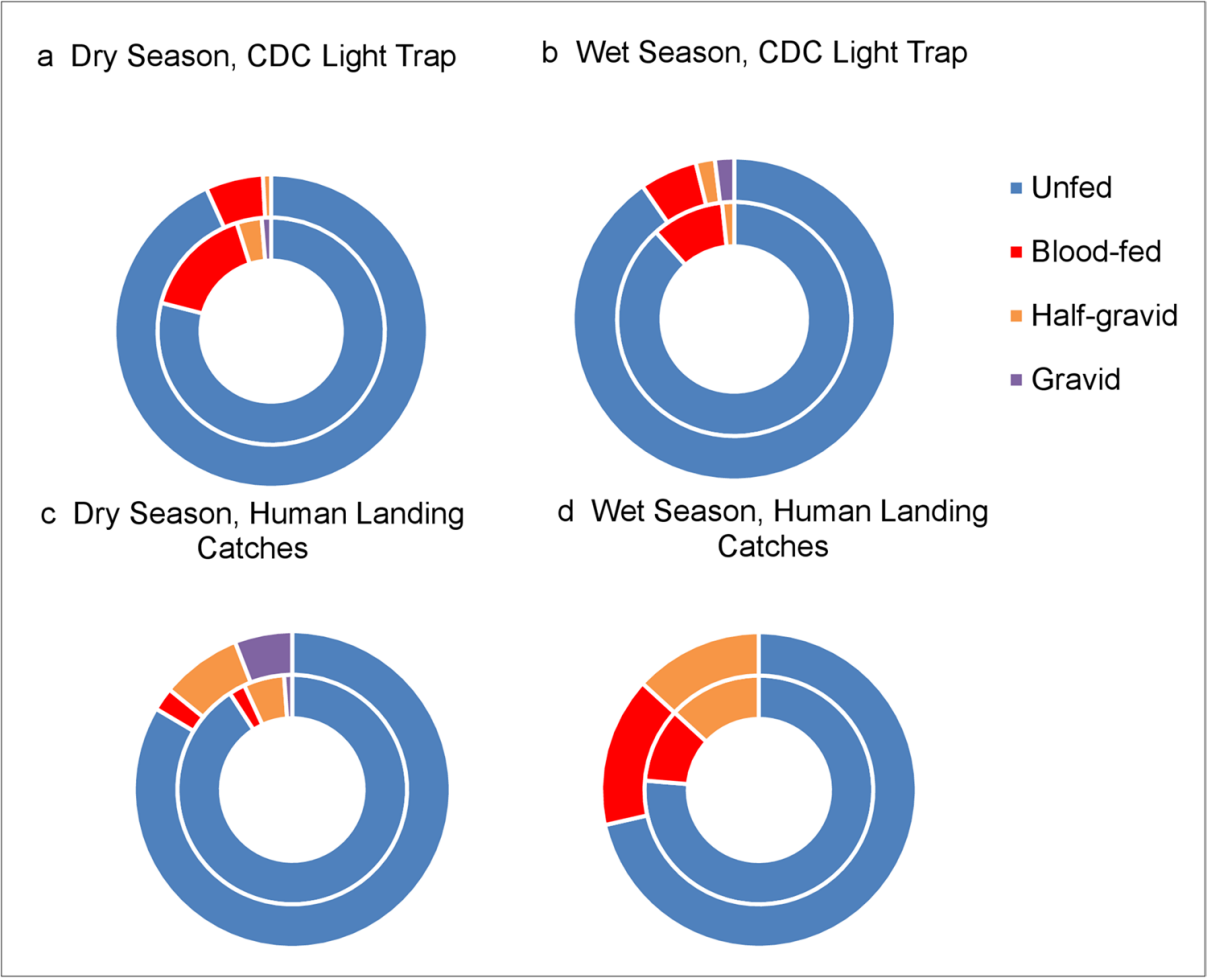
Gonotrophic status of female *An*. *arabiensis* mosquitoes collected using human landing catches (HLCs) and CDC light traps indoor and outdoor in irrigated zones in 2019. Outer rings referred to outdoor collection; inner rings referred to indoor collection

The gonotrophic status of *An*. *arabiensis* variation was significantly higher indoor compared to outdoor collections using CDC light trap during the dry season (*χ*^2^ = 11.94, df = 3, *P* = 0.03). In contrast, there was no significant difference during the wet season (*χ*^2^ = 3.08, df = 3, *P* = 0.38) (Fig. 5). There was also no significant difference in the gonotrophic status of *An*. *arabiensis* between indoor and outdoor collections using HLC during the dry (*χ*^2^ = 5.68, df = 3, *P* = 0.13) and wet seasons (*χ*^2^ = 1.11, df = 3, *P* = 0.78) (Fig. 5). Most of the *An*. *arabiensis* collected by HLC and CDC light trap were unfed (Fig. 5). Due to the small number of mosquito collections in HLC and CDC light traps in non-irrigated zone, the gonotrophic status was not analyzed.

**Fig. 6:**
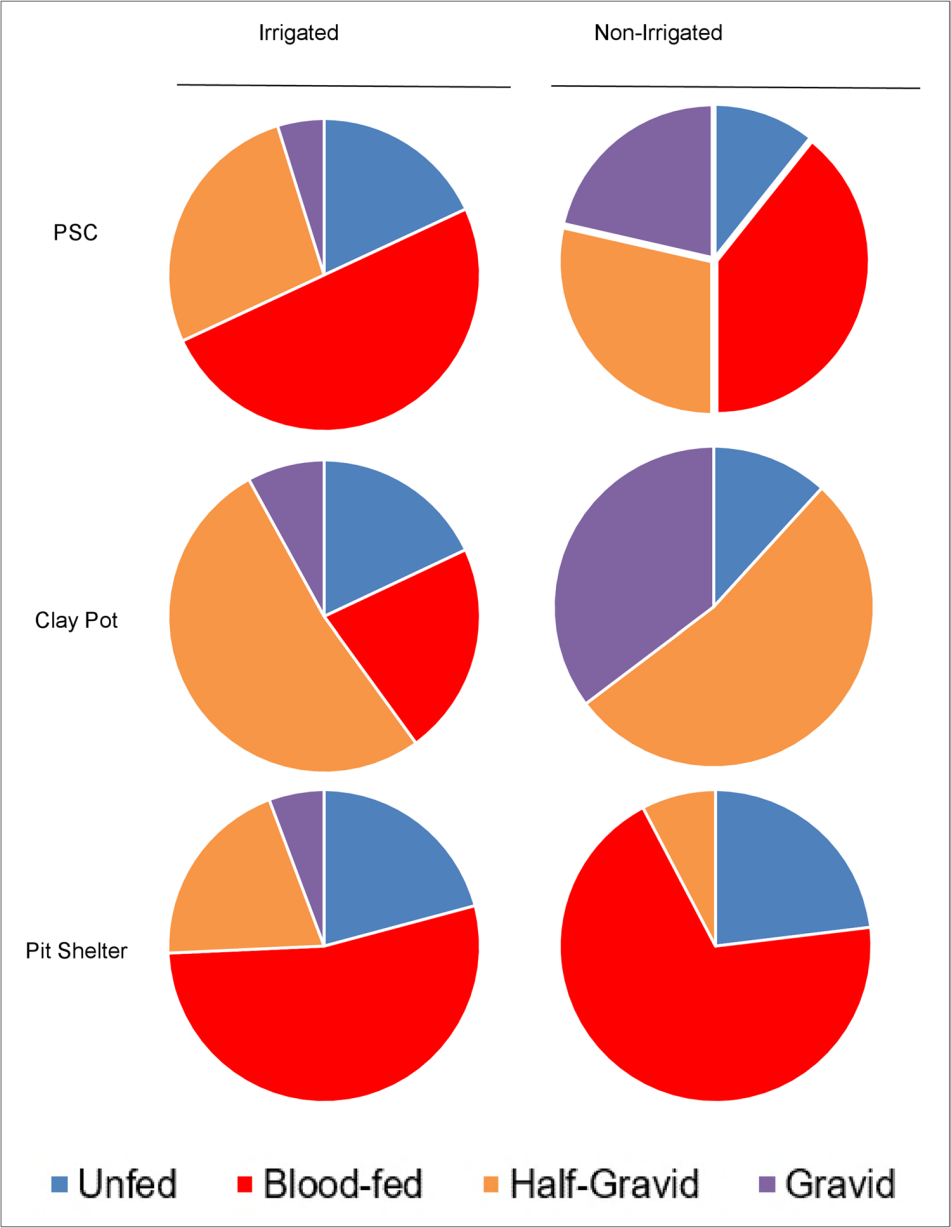
Gonotrophic status of resting female *An*. *arabiensis* mosquitoes collected using pyrethrum spray catches (PSCs) (indoor), clay pot (outdoor), and pit shelter (outdoor) from irrigated and non-irrigated zones in 2019

The gonotrophic status of *An*. *arabiensis* variation was significantly higher in the irrigated zone than in the non-irrigated zone in PSC (*χ*^2^ = 10.51, df = 3, *P* = 0.02) and clay pot (*χ*^2^ = 14.64, df = 3, *P* = 0.01) collections whereas there was no significant difference in pit shelter (*χ*^2^ = 6.87, df = 3, *P* = 0.08) collections (Fig. 6). Pit shelters and PSC yielded a higher proportion of blood-fed *An*. *arabiensis* compared to clay pots that captured mostly half-gravid *An*. *arabiensis* (Fig. 6).

#### Blood meal indices

The majority of the blood meals were of bovine origin (71.6%). Only 0.6% of mosquito samples had human blood meals and less than 1% of the samples had blood meals of goat, pig, or dog origin (Table 2). In the irrigated zone, outdoor HBI of *An*. *arabiensis* was almost twofold higher (outdoor, 5.20%; indoor, 3.90%) than indoor whereas in the non-irrigated zone, the blood meals were of bovine origin (Table 2).

**Table 2:** The host feeding preference of *An. arabiensis* mosquitoes collected indoor and outdoor by different collection methods from irrigated and non-irrigated zones from seasonal sampling in 2019

Blood-meal origins	Indoor		Outdoor			
	PSC^a^ (%)		Clay pot (%)		Pit shelter (%)	
	Irrigated zone	Non-irrigated zone	Irrigated zone	Non-irrigated zone	Irrigated zone	Non-irrigated zone
No. tested	205	13	114	1	136	6
Human	2 (1.0)	0 (0)	1 (0.9)	0 (0)	0 (0)	0 (0)
Bovine	157 (76.6)	10 (76.9)	67 (58.8)	1 (100.0)	101 (74.3)	4 (66.7)
Human + bovine	4 (2.0)	0 (0)	0 (0)	0 (0)	8 (5.9)	0 (0)
Human + dog	2 (1.0)	0 (0)	0 (0)	0 (0)	0 (0)	0 (0)
Bovine + dog	1 (0.5)	0 (0)	1 (0.9)	0 (0)	0 (0)	0 (0)
Pig	1 (0.5)	0 (0)	0 (0)	0 (0)	0 (0)	0 (0)
Human + goat	0 (0)	0 (0)	1 (0.9)	0 (0)	0 (0)	0 (0)
Human + bovine + dog	0 (0)	0 (0)	2 (1.8)	0 (0)	0 (0)	0 (0)
Human + dog + goat	0 (0)	0 (0)	1 (0.9)	0 (0)	0 (0)	0 (0)
Goat	0 (0)	0 (0)	2 (1.8)	0 (0)	0 (0)	0 (0)
Dog	0 (0)	0 (0)	0 (0)	0 (0)	1 (0.7)	0 (0)
Bovine + goat	0 (0)	0 (0)	0 (0)	0 (0)	3 (2.2)	0 (0)
Bovine + pig	0 (0)	0 (0)	1 (0.9)	0 (0)	0 (0)	0 (0)
Unknown	38 (18.5)	3 (23.1)	38 (33.3)	0 (0)	23 (16.9)	2 (33.3)
HBI^b^	3.90%	0.00%	4.39%	0.00%	5.88%	0.00%

^a^*PSC*, pyrethrum spray catches

^b^Human blood index (HBI) was calculated as the number of mosquito positive for human blood meal (including mixed blood meal) divided by the total number tested

#### Sporozoite rate and entomological inoculation rate

Sporozoite-positive *An*. *arabiensis* samples were detected in the irrigation zone only and the sporozoite rate was twofold higher indoors than outdoors (Table 3). None of the *An*. *funestus* samples tested positive for sporozoites (Table 3). The annual EIR for *An*. *arabiensis* in irrigated zone was 0.71 infective bites/person/year (ib/p/year) and was higher indoors than outdoors (Table 4). Malaria transmission was not detected in the non-irrigated zone (Table 4).

**Table 3:** The sporozoite rate of *An. arabiensis* and *An*. *funestus* mosquitoes collected indoor and outdoor by different collection methods from irrigated and non-irrigated zones in 2019

Zone	Location	Method	*An*. *arabiensis*			*An*. *funestus*		
			Mosquito tested	No. positive	Sporozoite rate (%)	Mosquito tested	No. positive	Sporozoite rate (%)
Irrigated zone	Indoors	PSC^a^	170	5	2.9	1	0	0
		CDC LT^b^	228	1	0.4	2	0	0
		HLC^c^	246	4	1.6	11	0	0
		Sub-total	644	10	1.6	14	0	0
	Outdoors	Clay pot	238	3	1.3	1	0	0
		Pit shelter	198	0	0	1	0	0
		CDC LT	101	1	1	1	0	0
		HLC	150	2	1.3	1	0	0
		Sub-total	687	6	0.8	4	0	0
Non-irrigated zone	Indoors	PSC	14	0	0	0	0	-
		CDC LT	8	0	0	0	0	-
		HLC	3	0	0	0	0	-
		Sub-total	25	0	0	0	0	-
	Outdoors	Clay pot	18	0	0	0	0	-
		Pit shelter	24	0	0	2	0	0
		CDC LT	5	0	0	0	0	-
		HLC	5	0	0	0	0	-
		Sub-total	52	0	0	2	0	0

^a^*PSC*, pyrethrum spray catches

^b^*CDC LT*, CDC light trap

^c^*HLC*, human landing catches

- Not tested

**Table 4:** The annual entomological inoculation rate (EIR) of *Anopheles* mosquitoes sampled in irrigated and non-irrigated zones in 2019

Study site and species	Indoors				Outdoors			
	HBI^a^ (%)	Sporozoite rate (%)	Mosquito density	Annual EIR	HBI (%)	Sporozoite rate (%)	Mosquito density	Annual EIR
Irrigated zone								
*An*. *arabiensis*	3.90	1.60	3.58	0.41	5.20	0.80	3.96	0.30
*An*. *funestus*	-	0.00	0.04	-	-	0.00	0.02	-
Non-irrigated zone								
*An*. *arabiensis*	0.00	0.00	0.14	0.00	0.00	0.00	0.21	0.00
*An*. *funestus*	-	-	0.00	-	-	0.00	0.01	-

^a^*HBI*, human biting index

- Not tested

### Longitudinal surveillance

**Fig. 7:**
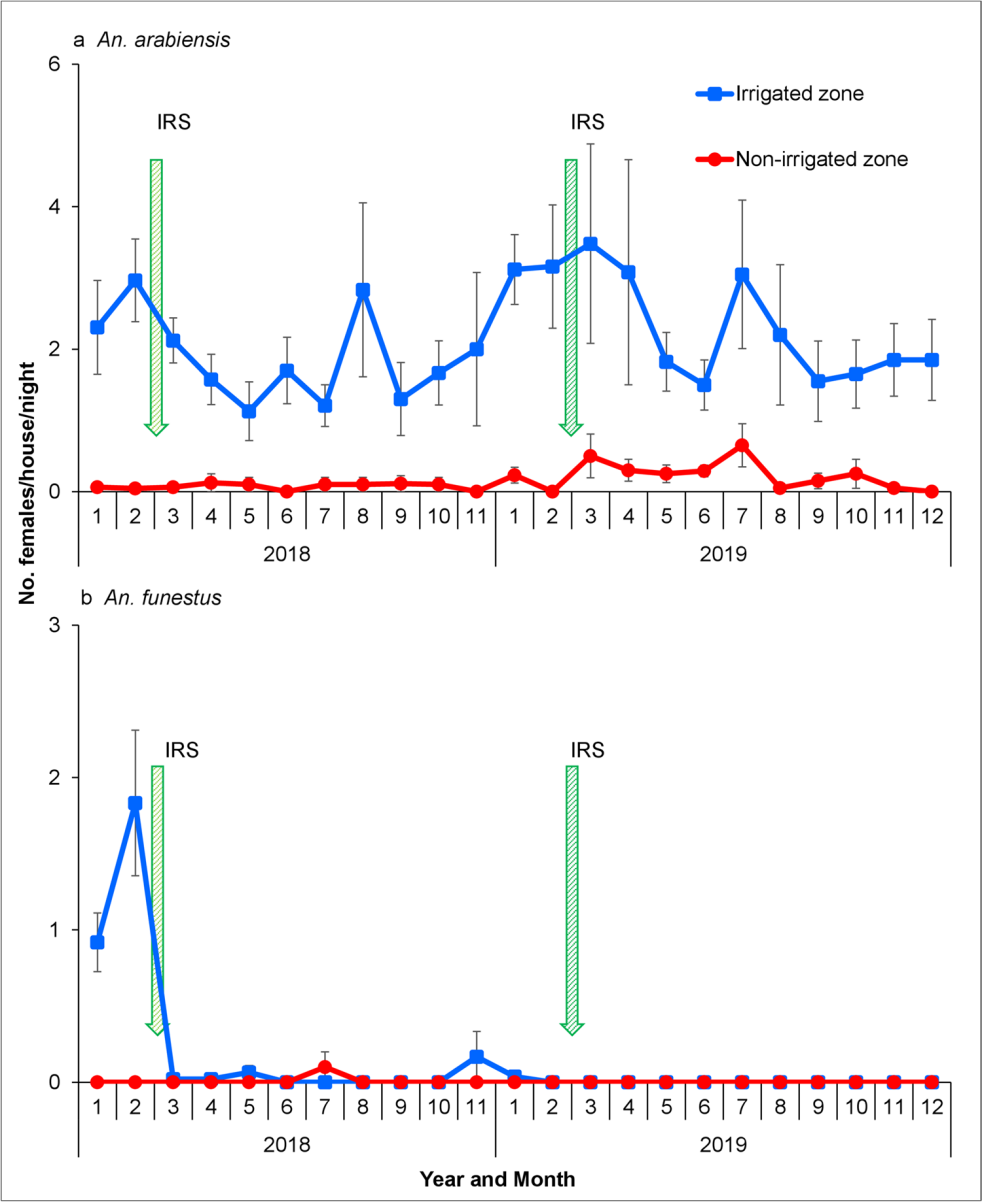
Indoor resting density of female **a** *An*. *arabiensis* and **b** *An*. *funestus* mosquitoes collected using pyrethrum spray catches. Error bars were for the standard error for the *Anopheles* mosquitoes collected. Abbreviations: IRS, indoor residual spraying

A total of 2,474 female anophelines were collected between January 1st, 2018, and December 31st, 2019, consisting of 2,248 (90.9%) *An*. *gambiae* s.l., 225 (9.1%) *An*. *funestus*, and 1 (0.04%) *An*. *coustani*. 760 specimens (621 *An*. *gambiae* s.l. and 139 *An*. *funestus*) were analyzed for sibling species identification. For the *An*. *gambiae* s.l. specimens, PCR results indicated that 99.7% were *An*. *arabiensis* and 0.3% *An*. *gambiae* s.s. All the *An*. *funestus* subjected to species identification were confirmed as *An*. *funestus* s.s. Overall, *An*. *arabiensis* was the dominant vector of malaria in the study sites. After adjusting for month, *Anopheles arabiensis* indoor resting density was 2.19 in irrigated zone and significantly higher than 0.21 in the non-irrigated zone (*Z* =  − 4.690, df = 1540, *P* < 0.001) (Fig. 7, Additional file 1: Table S4). The difference in indoor resting density of *An*. *arabiensis* during the dry and wet seasons was not significant (*Z* =  − 1.055, df = 1540, *P* = 0.292) (Additional file 1: Table S4). The *An*. *funestus* indoor resting density was 0.23 in irrigated zone while only few *An*. *funestus* were collected in the non-irrigated zone (Fig. 7). The study clearly indicated that the malaria vector species were more abundant in the irrigated zone than in the non-irrigated zone. In the irrigated zone, the HBI of *An*. *funestus* (49.43%) was 14-fold higher than *An*. *arabiensis* (3.44%) whereas in the non-irrigated zone, none of the *An*. *arabiensis* tested positive for human blood (Table 5).

**Table 5:** Host feeding preference of *An. arabiensis* and *An. funestus* mosquitoes collected indoors using pyrethrum spray catches from irrigated and non-irrigated zone from longitudinal sampling in 2018 and 2019

Blood-meal origins	*An. arabiensis* (%)		*An. funestus* (%)	
	Irrigated zone	Non-irrigated zone	Irrigated zone	Non-irrigated zone
No. tested	494	27	87	0
Human	7 (1.6)	0 (0)	39 (44.8)	0 (0)
Bovine	390 (78.8)	22 (81.5)	30 (34.5)	0 (0)
Human + bovine	5 (1.0)	0 (0)	3 (3.4)	0 (0)
Human + dog	4 (0.8)	0 (0)	0 (0)	0 (0)
Bovine + dog	4 (0.8)	0 (0)	0 (0)	0 (0)
Pig + dog	3 (0.6)	0 (0)	0 (0)	0 (0)
Goat	2 (0.4)	0 (0)	0 (0)	0 (0)
Dog	2 (0.4)	0 (0)	0 (0)	0 (0)
Bovine + goat	2 (0.4)	0 (0)	0 (0)	0 (0)
Pig	1 (0.2)	1 (3.7)	1 (1.1)	0 (0)
Human + bovine + pig	1 (0.2)	0 (0)	0 (0)	0 (0)
Bovine + dog + pig	1 (0.2)	0 (0)	0 (0)	0 (0)
Human + pig	0 (0)	0 (0)	1 (1.1)	0 (0)
Unknown	72 (14.5)	4 (14.8)	13 (14.9)	0 (0)
HBI^a^	3.44%	0.00%	49.43%	0.00%

^a^Human blood index (HBI) was calculated as the number of mosquito positive for human blood meal (including mixed blood meal) divided by the total number tested

## Discussion

This study investigated the effect of concrete canal and flood irrigation systems on species composition, malaria vector abundance and their seasonality, vector behavior, and malaria transmission in irrigated and non-irrigated sites where vector control using LLINs and IRS was being undertaken during the study period. Findings of the study demonstrated that *An*. *arabiensis* was the dominant anopheline species and was more abundant in irrigated zone compared to the non-irrigated zone. The high vector density of *An*. *arabiensis* together with their potential to transmit *P*. *falciparum* confirms the significant risk of malaria transmission in populations living within the irrigation scheme. The secondary aim of the study was to compare the trap effectiveness of CDC light traps against the gold standard, HLC. The results indicated that CDC light traps were equally effective in sampling malaria vectors as the HLC. This indicates the usefulness of this tool for continuous operational surveillance of the mosquito vectors within the study site.

The distribution of *An*. *arabiensis* is generally concentrated in the drier savannah environments where rainfall is < 1000 mm (Coetzee et al. 2000). *Anopheles funestus* was scarcely collected in the study site. Other anopheline species that were reported, and occurred only in the irrigated zone, were *An*. *coustani* and *An*. *pharoensis*. These two mosquito species have previously been considered as secondary vectors of malaria in Africa (Afrane et al. 2016) but recent studies have shown that they could play an important role in malaria transmission (Kerah-Hinzoumbé et al. 2009; Kibret et al. 2012; Mwangangi et al. 2013). Thus, it is prudent to integrate them in malaria vector surveillance and control strategies particularly where they are abundant.

A significant variation in vector density was observed in the irrigated and non-irrigated zones which is consistent with previous studies that the introduction of irrigation schemes leads to an increase in vector density and abundance (Ijumba et al. 2002a; Briet et al. 2003; Diuk-Wasser et al. 2005; Muturi et al. 2008b). In the irrigated zone, the irrigated canals, seepage areas, and flooded irrigated fields serve as the main larval habitats and provide stable mosquito breeding habitats during the dry season when other larval habitats dry up. In contrast, the low *An*. *arabiensis* density in the non-irrigated zone may be due to the temporary and parched nature of aquatic habitats (rain pools, rice fields, and edges of seasonal swamps) during the dry season. These observations have also been reported in similar studies in the Mwea Irrigation Scheme and the neighboring non-irrigated agroecosystems (Muturi et al. 2006). The indoor resting density of *An*. *funestus* was generally low; however, their indoor abundance was relatively high during the dry season of 2018 in the irrigated zone prior to the application of the IRS in the study site. Thereafter, *An*. *funestus* was rarely collected from our study. This can be attributed to the application of Actellic® 300CS IRS which has been shown to significantly reduce the indoor resting density of *An*. *funestus* in the same area (Abong’o et al. 2020). Indoor residual spraying is known to be highly effective on endophilic and anthropophilic mosquito species such as *An*. *funestus* due to high exposure to the wall sprayed insecticides.

The indoor and outdoor host-seeking density of *An*. *arabiensis* varied significantly with the highest biting densities collected indoors. Studies conducted over three decades ago by Githeko et al. showed that *An*. *arabiensis* was more likely to bite indoor than outdoor before the scale up of vector control intervention in western Kenya (Githeko et al. 1994a, 1996). In the present study, the endophagic tendency of *An*. *arabiensis* was still observed despite the high LLINs coverage and application of IRS in the study sites. This could be attributed to behavior of this species whereby it may enter a house protected with malaria vector control interventions in search of unprotected host, but exit without fatal exposure to insecticide-treated surfaces (Kitau et al. 2012; Okumu et al. 2013; Asale et al. 2014). Nonetheless, outdoor biting behavior was observed for this mosquito species which is consistent with other studies within its distribution range (Gatton et al. 2013).

It is worth mentioning that when comparing the traps deployed in the study, the mean vector density varied significantly between traps. The outdoor density of *An*. *arabiensi*s was significantly higher in pit shelters and clay pots than for CDC light traps; in contrast, the indoor density of *An*. *arabiensis* was not significantly different between traps. Such variations were likely driven by differences in vector behavior, vector species composition, and history of malaria interventions rather than differences in the efficiency between the traps. Wide variation in the vector density of each trapping method has also been observed by Degefa et al. in a study conducted in western and attributed this variations to vector behavior (Degefa et al. 2019).

Human landing catches have been considered the gold standard method for estimating mosquito-human contact. However, it is a labor-intensive procedure requiring highly trained collectors, extensive supervision, variation in the skill of the collectors or their individual attractiveness to mosquitoes, and ethical concerns associated with potential exposure to infectious mosquito bites (Knols et al. 1995; WHO 2013). Our results indicate that CDC light trap is an effective trapping alternative to HLC for continuous operational surveillance of mosquito vectors within the study sites. In a recent study conducted in western Kenya and southwestern Ethiopia, human-odor-baited CDC light traps (HBLT) collected twice the number of outdoor host-seeking *An*. *arabiensis* and *An*. *funestus* compared to non-baited CDC light traps (Degefa et al. 2020). Thus, it will be important to evaluate the effectiveness of this tool in the study sites that could be a better outdoor surveillance tool than the non-baited CDC light trap.

Anthropophily was highest in *An*. *funestus* compared to *An*. *arabiensis* in the irrigated zone. These findings are consistent with previous studies that have reported *An*. *funestus* s.s. to exhibit anthropophagic behavior in Kenya (Githeko et al. 1994b; Mwangangi et al. 2003) and in other parts of Africa (Tanga et al. 2011; Mzilahowa et al. 2012; Dadzie et al. 2013). However, in recent reports, they have been shown to also feed on bovine (Degefa et al. 2017; Ogola et al. 2018) in the presence of LLINs. This plasticity of the feeding behavior of the vector may influence malaria transmission, leading to residual transmission after the densities of endophilic and endophagic vectors have been reduced by the interventions (Durnez and Coosemans 2013; Afrane et al. 2016). The life histories of *An*. *arabiensis* population of southern Tanzania were simulated in a model by Killeen et al. and estimated that two-thirds of the vector feeds outdoor in an area where bednet usage is high (Killeen et al. 2016). Studies have indicated that *An*. *arabiensi*s exhibits behavior that mediates residual transmission such as feeding outdoors on humans or cattle and rapidly exiting houses without fatal exposure to insecticide-treated surfaces (Killeen et al. 2016). Findings of the present study demonstrated that *An*. *arabiensis* fed on humans both indoors and outdoors with a higher HBI outdoors and predominantly fed on bovine. However, it remains capable of transmitting malaria whenever it can feed on humans.

There was a significant difference in the risk of malaria transmission by *An*. *arabiensis* in the two zones, with higher transmission risk in the irrigated zone. These results show that irrigation has an effect on malaria transmission and *An*. *arabiensis* played a significant role in transmission. In addition, this species contributed almost equally to both indoor and outdoor transmission. In many studies, irrigated areas have been associated with increased malaria transmission than neighboring non-irrigated areas (Oomen et al. 1988; Gbakima 1994; Yohannes et al. 2005); however, in some cases, introduction of irrigation schemes reduces (Ijumba et al. 2002a; Sissoko et al. 2004) or has no impact on malaria transmission (Faye et al. 1995). Hence, the impact of water development projects on malaria transmission is variable and the transmission dynamic likely depends on the local epidemiological setting. Our data also suggest that the zoophagic behavior of *An*. *arabiensis* could be accounting for the low transmission in the irrigated zone whereas the low vector densities limited transmission in the non-irrigated zone. The zoophagic tendency of *An*. *arabiensis* indicates zooprophylaxis may be a potential strategy for malaria control.

The limitation of our study is the lack of information on the movement of endophagic mosquitoes as they exit the house after feeding and/or resting. This information would have improved the understanding of the effect of insecticide-based vector control interventions in the houses on the normal movement, density, and reticence feeding of endophilic species (WHO 1975; Müller et al. 2017).

## Conclusion

*Anopheles arabiensis* was the dominant vector of malaria in the study sites. Our study demonstrated that there is a difference in malaria transmission by *An*. *arabiensis* between the two zones with higher transmission risk in the irrigated zone and the reason attributing to this is the high vector densities in the irrigated zone. The density of *An*. *funestus* was generally low nonetheless the anthropophily was highest in *An*. *funestus* compared to *An*. *arabiensis*. While most of the malaria transmission by *An*. *arabiensis* occurred indoors, transmission also occurred outdoors. The irrigation scheme should therefore incorporate additional vector management strategies to complement the LLINs and IRS to control outdoor malaria transmission. Larval source management to reduce vector density and new tools for protecting human exposed outdoor will probably be needed to control outdoor seeking mosquitoes. This is among the first few studies conducted in this newly established irrigation scheme in western Kenya and the findings will guide the Ministry of Health, Ministry of Agriculture, Livestock, Fisheries and Irrigation, and Ministry of Environment and Forestry in developing strategies that promote crop production in irrigated areas while limit proliferation of mosquito vector populations.

## Supplementary Information

Below is the link to the electronic supplementary material.

Supplementary file1 (DOCX 17.5 KB)

Supplementary file2 (DOCX 19.2 KB)

Supplementary file3 (DOCX 20 KB)

Supplementary file4 (DOCX 16.9 KB)

## Data Availability

All data generated or analyzed during this study are included in this published article [and its supplementary information files].

## Abbreviations

AIC: Akaike information criterion
CDC: Centers for Disease Control and Prevention
DNA: Deoxyribonucleic acid
EIRs: Entomological inoculation rates
HBI: Human biting index
HLCs: Human landing catches
ICEMR: International Center of Excellence for Malaria Research
IRS: Indoor residual spraying
LLINs: Long-lasting insecticidal nets
PCR: Polymerase chain reaction
PSCs: Pyrethrum spray catches
qPCR: Quantitative polymerase chain reaction
RNA: Ribonucleic acid
s.l.: Sensu lato
s.s.: Sensu stricto
WHO: World Health Organization
