# Supplementary material for: Malaria vector bionomics and transmission in irrigated and non-irrigated sites in western Kenya
Source: Parasitol Res. 2022 Oct 7;121(12):3529–45. doi: 10.1007/s00436-022-07678-2 (PMC9653358; doi:10.1007/s00436-022-07678-2)
Supplement: Supplementary file 1 — Supplementary file1 (DOCX 17.5 KB) [file 436_2022_7678_MOESM1_ESM.docx]

**Additional file 1: Table S1** Negative binomial mixed model and linear model analysis of differences of outdoor density of female *An*. *arabiensis* by different zones and collection methods

| **Model Number** | **Model Type** | **Random Variables** | **Fixed Variables/ Coefficients** | **Estimate** | **S.E.^a^** | **z** | ***p*** | **AIC^b^** |
| --- | --- | --- | --- | --- | --- | --- | --- | --- |
| 1a | Linear | - | intercept | 0.59468 | 0.22519 | 2.641 | 0.00827 | 2187.7 |
|  |  |  | zone | -2.96056 | 0.15696 | -18.862 | <0.001 |  |
|  |  |  | season | -0.12396 | 0.12795 | -0.969 | 0.33264 |  |
|  |  |  | method clay pot | 0.32042 | 0.18108 | 1.770 | 0.07681 |  |
|  |  |  | method hlc^c^ | 0.04487 | 0.21294 | 0.211 | 0.83311 |  |
|  |  |  | method pit shelter | 1.73790 | 0.20254 | 8.581 | <0.001 |  |
|  |  |  | bed nets | 0.09364 | 0.08921 | 1.050 | 0.29384 |  |
| 1b | Linear | - | intercept | 0.706091 | 0.150007 | 4.707 | <0.001 | 2185.6 |
|  |  |  | zone | -2.985425 | 0.155831 | -19.158 | <0.001 |  |
|  |  |  | method clay pot | 0.293152 | 0.178674 | 1.641 | 0.101 |  |
|  |  |  | method hlc | -0.006161 | 0.209500 | -0.029 | 0.977 |  |
|  |  |  | method pit shelter | 1.738454 | 0.195200 | 8.906 | <0.001 |  |
| 1c | Linear | - | intercept | 1.37604 | 0.07992 | 17.22 | <0.001 | 2304.9 |
|  |  |  | zone | -2.94058 | 0.15597 | -18.85 | <0.001 |  |
| 2^d^ | NBMM^e^ | house number, cluster | intercept | -0.007683 | 0.306343 | -0.025 | 0.97999 | 2150.2 |
|  |  |  | zone | -2.852747 | 0.344714 | -8.276 | <0.001 |  |
|  |  |  | method clay pot | 0.663984 | 0.213009 | 3.117 | 0.00183 |  |
|  |  |  | method hlc | 0.257399 | 0.266529 | 0.966 | 0.33417 |  |
|  |  |  | method pit shelter | 2.052270 | 0.319039 | 6.433 | <0.001 |  |
| 3 | NBMM | cluster | intercept | 1.3267 | 0.3059 | 4.338 | <0.001 | 2280.4 |
|  |  |  | zone | -3.0253 | 0.4356 | -6.944 | <0.001 |  |
| 4 | NBMM | date | intercept | 1.0777 | 0.1623 | 6.642 | <0.001 | 2228.4 |
|  |  |  | zone | -2.9237 | 0.1659 | -17.625 | <0.001 |  |
| 5 | NBMM | house number | intercept | 0.6267 | 0.1336 | 4.692 | <0.001 | 2191.4 |
|  |  |  | zone | -2.8812 | 0.2314 | -12.454 | <0.001 |  |
| 6 | NBMM | date:house number, house number | intercept | 0.1833 | 0.1326 | 1.382 | 0.167 | 2192.7 |
|  |  |  | zone | -2.9049 | 0.2364 | -12.290 | <0.001 |  |
| 7 | NBMM | date:cluster, cluster | intercept | 1.3056 | 0.3172 | 4.116 | <0.001 | 2206.0 |
|  |  |  | zone | -3.1951 | 0.4671 | -6.840 | <0.001 |  |
| 8 | NBMM | house number:(date:cluster), date:cluster, cluster | intercept | 1.0277 | 0.2993 | 3.434 | 0.000596 | 2219.6 |
|  |  |  | zone | -3.3375 | 0.4457 | -7.489 | <0.001 |  |

^a^ S.E., standard error

^b^ AIC, akaike information criterion

^c^ hlc, human landing catches

^d^ Best model selected with the lowest AIC

^e^ NBMM, negative binomial mixed model
