# Supplementary material for: Malaria vector bionomics and transmission in irrigated and non-irrigated sites in western Kenya
Source: Parasitol Res. 2022 Oct 7;121(12):3529–45. doi: 10.1007/s00436-022-07678-2 (PMC9653358; doi:10.1007/s00436-022-07678-2)
Supplement: Supplementary file 2 — Supplementary file2 (DOCX 19.2 KB) [file 436_2022_7678_MOESM2_ESM.docx]

**Additional file 1: Table S2** Negative binomial mixed model and linear model analysis of differences of indoor density of female *An*. *arabiensis* by different zones and collection methods

| **Model Number** | **Model Type** | **Random Variables** | **Fixed Variables/ Coefficients** | **Estimate** | **S.E.^a^** | **z** | ***p*** | **AIC^b^** |
| --- | --- | --- | --- | --- | --- | --- | --- | --- |
| 1 | Linear | - | intercept | 1.16622 | 0.24955 | 4.673 | <0.001 | 1755.1 |
|  |  |  | zone | -3.18213 | 0.18820 | -16.908 | <0.001 |  |
|  |  |  | season | -0.21207 | 0.15338 | -1.383 | 0.167 |  |
|  |  |  | method hlc^c^ | -0.11818 | 0.22001 | -0.537 | 0.591 |  |
|  |  |  | method psc^d^ | -0.01193 | 0.18646 | -0.064 | 0.949 |  |
|  |  |  | bed nets | 0.15185 | 0.11031 | 1.377 | 0.169 |  |
| 2 | Linear | - | intercept | 1.264555 | 0.252769 | 5.003 | <0.001 | 1752.9 |
|  |  |  | zone | -3.131203 | 0.188088 | -16.648 | <0.001 |  |
|  |  |  | season | -0.174899 | 0.153513 | -1.139 | 0.2546 |  |
|  |  |  | method hlc | -0.098923 | 0.218833 | -0.452 | 0.6512 |  |
|  |  |  | method psc | -0.007842 | 0.186437 | -0.042 | 0.9665 |  |
|  |  |  | bed nets | 0.283956 | 0.127985 | 2.219 | 0.0265 |  |
|  |  |  | occupants | -0.098207 | 0.048360 | -2.031 | 0.0423 |  |
| 3 | Linear | - | intercept | 1.36298 | 0.15748 | 8.655 | <0.001 | 1754.1 |
|  |  |  | zone | -3.21769 | 0.18950 | -16.980 | <0.001 |  |
|  |  |  | method hlc | -0.19691 | 0.21771 | -0.904 | 0.366 |  |
|  |  |  | method psc | -0.07882 | 0.18724 | -0.421 | 0.674 |  |
| 4 | Linear | - | intercept | 1.219525 | 0.248001 | 4.917 | <0.001 | 1752.1 |
|  |  |  | zone | -3.159974 | 0.189361 | -16.688 | <0.001 |  |
|  |  |  | method hlc | -0.110919 | 0.220365 | -0.503 | 0.6147 |  |
|  |  |  | method psc | -0.007934 | 0.187786 | -0.042 | 0.9663 |  |
|  |  |  | bed nets | 0.284947 | 0.128998 | 2.209 | 0.0272 |  |
|  |  |  | occupants | -0.105015 | 0.048355 | -2.172 | 0.0299 |  |
| 5 | Linear | - | intercept | 1.17307 | 0.19572 | 5.994 | <0.001 | 1748.5 |
|  |  |  | zone | -3.15499 | 0.18913 | -16.681 | <0.001 |  |
|  |  |  | bed nets | 0.29522 | 0.12672 | 2.330 | 0.0198 |  |
|  |  |  | occupants | -0.10578 | 0.04817 | -2.196 | 0.0281 |  |
| 6 | NBMM^e^ | house number, cluster | intercept | 1.02809 | 0.45856 | 2.242 | 0.0250 | 1718.5 |
|  |  |  | zone | -3.10285 | 0.46602 | -6.658 | <0.001 |  |
|  |  |  | method hlc | -0.39704 | 0.32712 | -1.214 | 0.2248 |  |
|  |  |  | method psc | -0.20615 | 0.26353 | -0.782 | 0.4340 |  |
|  |  |  | bed nets | 0.20595 | 0.14552 | 1.415 | 0.1570 |  |
|  |  |  | occupants | -0.14435 | 0.05652 | -2.554 | 0.0107 |  |
| 7 | NBMM | cluster | intercept | 1.16839 | 0.43206 | 2.704 | 0.00685 | 1747.3 |
|  |  |  | zone | -3.24786 | 0.45903 | -7.075 | <0.001 |  |
|  |  |  | method hlc | -0.10596 | 0.22371 | -0.474 | 0.63574 |  |
|  |  |  | method psc | -0.01901 | 0.23068 | -0.082 | 0.93433 |  |
|  |  |  | bed nets | 0.25053 | 0.14628 | 1.713 | 0.08676 |  |
|  |  |  | occupants | -0.09022 | 0.04854 | -1.859 | 0.06304 |  |
| 8 | NBMM | date | intercept | 1.0546 | 0.1671 | 6.311 | <0.001 | 1720.3 |
|  |  |  | zone | -3.3623 | 0.3045 | -11.041 | <0.001 |  |
| 9^f^ | NBMM | house number, date | intercept | 0.71118 | 0.36250 | 1.962 | 0.0498 | 1695.2 |
|  |  |  | zone | -3.12624 | 0.33248 | -9.403 | <0.001 |  |
|  |  |  | method hlc | -0.32106 | 0.31501 | -1.019 | 0.3081 |  |
|  |  |  | method psc | -0.05661 | 0.32143 | -0.176 | 0.8602 |  |
|  |  |  | bednets | 0.21325 | 0.13769 | 1.549 | 0.1214 |  |
|  |  |  | occupants | -0.12140 | 0.05393 | -2.251 | 0.0244 |  |
| 10 | NBMM | date, cluster | intercept | 0.92978 | 0.30756 | 3.023 | 0.0025 | 1725.7 |
|  |  |  | zone | -3.32529 | 0.30402 | -10.938 | <0.001 |  |
|  |  |  | method hlc | -0.02995 | 0.21002 | -0.143 | 0.8866 |  |
|  |  |  | method psc | 0.10860 | 0.31112 | 0.349 | 0.7270 |  |
|  |  |  | bednets | 0.23813 | 0.13432 | 1.773 | 0.0763 |  |
| 11 | NBMM | house number, date | intercept | 0.57564 | 0.29126 | 1.976 | 0.0481 | 1692.4 |
|  |  |  | zone | -3.10603 | 0.32867 | -9.450 | <0.001 |  |
|  |  |  | bednets | 0.23243 | 0.13591 | 1.710 | 0.0872 |  |
|  |  |  | occupants | -0.12340 | 0.05389 | -2.290 | 0.0220 |  |
| 12 | NBMM | house number, date | intercept | 0.96950 | 0.32177 | 3.013 | 0.00259 | 1695.6 |
|  |  |  | zone | -3.16163 | 0.33285 | -9.499 | <0.001 |  |
|  |  |  | method hlc | -0.39361 | 0.31024 | -1.269 | 0.20454 |  |
|  |  |  | method psc | -0.11529 | 0.32148 | -0.359 | 0.71987 |  |
|  |  |  | occupants | -0.08248 | 0.04749 | -1.737 | 0.08242 |  |
| 13 | NBMM | house number, date | intercept | 0.80760 | 0.25902 | 3.118 | 0.00182 | 1693.3 |
|  |  |  | zone | -3.14519 | 0.32912 | -9.556 | <0.001 |  |
|  |  |  | occupants | -0.08036 | 0.04740 | -1.695 | 0.09000 |  |

^a^ S.E., standard error

^b^ AIC, akaike information criterion

^c^ hlc, human landing catches

^d^ psc, pyrethrum spray catches

^e^ NBMM, negative binomial mixed model

^f^ Best model selected with the 3^rd^ lowest AIC and variables of interest
