# Supplementary material for: Malaria vector bionomics and transmission in irrigated and non-irrigated sites in western Kenya
Source: Parasitol Res. 2022 Oct 7;121(12):3529–45. doi: 10.1007/s00436-022-07678-2 (PMC9653358; doi:10.1007/s00436-022-07678-2)
Supplement: Supplementary file 3 — Supplementary file3 (DOCX 20 KB) [file 436_2022_7678_MOESM3_ESM.docx]

**Additional file 1: Table S3** Negative binomial mixed model and linear model analysis of differences of HLC and CDC light trap host-seeking density of female *An*. *arabiensis*

| **Model Number** | **Model Type** | **Random Variables** | **Fixed Variables/ Coefficients** | **Estimate** | **S.E.^a^** | **z** | ***p*** | **AIC^b^** |
| --- | --- | --- | --- | --- | --- | --- | --- | --- |
| 1 | Linear | - | intercept | 0.89747 | 0.24074 | 3.728 | 0.000193 | 1574.0 |
|  |  |  | zone | -3.78264 | 0.24212 | -15.623 | <0.001 |  |
|  |  |  | season | -0.29486 | 0.15530 | -1.899 | 0.057607 |  |
|  |  |  | method hlc^c^ | 0.01521 | 0.15976 | 0.095 | 0.924162 |  |
|  |  |  | bed nets | 0.23151 | 0.12435 | 1.862 | 0.062644 |  |
|  |  |  | occupants | 0.06244 | 0.05565 | 1.122 | 0.261876 |  |
|  |  |  | location | -0.50956 | 0.15212 | -3.350 | 0.000809 |  |
| 2 | Linear | - | intercept | 0.84088 | 0.20726 | 4.057 | <0.001 | 1573.5 |
|  |  |  | zone | -3.78951 | 0.24227 | -15.642 | <0.001 |  |
|  |  |  | bed nets | 0.24726 | 0.11731 | 2.108 | 0.035050 |  |
|  |  |  | occupants | 0.03718 | 0.05383 | 0.691 | 0.489793 |  |
|  |  |  | location | -0.52103 | 0.15370 | -3.390 | 0.000699 |  |
| 3 | NBMM^d^ | house number, cluster | intercept | 0.54096 | 0.37791 | 1.431 | 0.15230 | 1512.2 |
|  |  |  | zone | -3.54422 | 0.38894 | -9.112 | <0.001 |  |
|  |  |  | method hlc | 0.09948 | 0.28923 | 0.344 | 0.73087 |  |
|  |  |  | bed nets | 0.41517 | 0.16019 | 2.592 | 0.00955 |  |
|  |  |  | occupants | -0.12036 | 0.07180 | -1.676 | 0.09367 |  |
|  |  |  | location | -0.40592 | 0.14549 | -2.790 | 0.00527 |  |
| 4 | NBMM | cluster | intercept | 0.57493 | 0.34421 | 1.670 | 0.094864 | 1561.5 |
|  |  |  | zone | -3.77743 | 0.39842 | -9.481 | <0.001 |  |
|  |  |  | method hlc | 0.10956 | 0.16004 | 0.685 | 0.493596 |  |
|  |  |  | bed nets | 0.36899 | 0.14193 | 2.600 | 0.009331 |  |
|  |  |  | occupants | 0.02682 | 0.05565 | 0.482 | 0.629843 |  |
|  |  |  | location | -0.53787 | 0.14932 | -3.602 | 0.000316 |  |
| 5 | NBMM | house number | intercept | 0.54490 | 0.34686 | 1.571 | 0.11619 | 1511.1 |
|  |  |  | zone | -3.53826 | 0.32750 | -10.804 | <0.001 |  |
|  |  |  | method hlc | 0.08726 | 0.30119 | 0.290 | 0.77202 |  |
|  |  |  | bed nets | 0.40248 | 0.16225 | 2.481 | 0.01312 |  |
|  |  |  | occupants | -0.11901 | 0.07273 | -1.636 | 0.10175 |  |
|  |  |  | location | -0.39692 | 0.14569 | -2.724 | 0.00644 |  |
| 6 | NBMM | date | intercept | 0.43856 | 0.27557 | 1.591 | 0.1115 | 1529.6 |
|  |  |  | zone | -3.77494 | 0.33029 | -11.429 | <0.001 |  |
|  |  |  | method hlc | 0.13006 | 0.14799 | 0.879 | 0.3795 |  |
|  |  |  | bed nets | 0.25505 | 0.12890 | 1.979 | 0.0478 |  |
|  |  |  | occupants | 0.07472 | 0.05371 | 1.391 | 0.1642 |  |
|  |  |  | location | -0.59210 | 0.14032 | -4.220 | <0.001 |  |
| 7^e^ | NBMM | house number, date | intercept | 0.32559 | 0.34050 | 0.956 | 0.3390 | 1478.3 |
|  |  |  | zone | -3.56800 | 0.36257 | -9.841 | <0.001 |  |
|  |  |  | method hlc | 0.09417 | 0.26810 | 0.351 | 0.7254 |  |
|  |  |  | bed nets | 0.28635 | 0.14940 | 1.917 | 0.0553 |  |
|  |  |  | occupants | -0.02062 | 0.07123 | -0.290 | 0.7722 |  |
|  |  |  | location | -0.41860 | 0.13186 | -3.175 | 0.0015 |  |
| 8 | NBMM | house number, date, cluster | intercept | 0.32560 | 0.34050 | 0.956 | 0.3390 | 1480.3 |
|  |  |  | zone | -3.56801 | 0.36257 | -9.841 | <0.001 |  |
|  |  |  | method hlc | 0.09417 | 0.26811 | 0.351 | 0.7254 |  |
|  |  |  | bed nets | 0.28635 | 0.14940 | 1.917 | 0.0553 |  |
|  |  |  | occupants | -0.02062 | 0.07123 | -0.290 | 0.7722 |  |
|  |  |  | location | -0.41860 | 0.13186 | -3.175 | 0.0015 |  |

^a^ S.E., standard error

^b^ AIC, akaike information criterion

^c^ hlc, human landing catches

^d^ NBMM, negative binomial mixed model

^e^ Best model selected with the lowest AIC
