# Supplementary material for: Malaria vector bionomics and transmission in irrigated and non-irrigated sites in western Kenya
Source: Parasitol Res. 2022 Oct 7;121(12):3529–45. doi: 10.1007/s00436-022-07678-2 (PMC9653358; doi:10.1007/s00436-022-07678-2)
Supplement: Supplementary file 4 — Supplementary file4 (DOCX 16.9 KB) [file 436_2022_7678_MOESM4_ESM.docx]

**Additional file 1: Table S4** Negative binomial and linear models analysis of differences of indoor resting density of female *An*. *arabiensis* in irrigated and non-irrigated zones

| **Model Number** | **Model Type** | **Random Variables** | **Fixed Variables/ Coefficients** | **Estimate** | **S.E.^a^** | **z** | ***p*** | **AIC^b^** |
| --- | --- | --- | --- | --- | --- | --- | --- | --- |
| 1 | Linear | - | intercept | 0.91860 | 0.07586 | 12.110 | <0.001 | 4186.8 |
|  |  |  | zone | -2.34679 | 0.13092 | -17.925 | <0.001 |  |
|  |  |  | season | -0.34689 | 0.10826 | -3.204 | 0.00135 |  |
| 2 | Linear | - | intercept | -682.6637 | 220.5752 | -3.095 | 0.001969 | 4179.3 |
|  |  |  | zone | -2.4271 | 0.1331 | -18.235 | <0.001 |  |
|  |  |  | season | -0.3716 | 0.1088 | -3.416 | 0.000635 |  |
|  |  |  | year | 0.3387 | 0.1093 | 3.099 | 0.001941 |  |
| 3 | NBMM^c^ | cluster | intercept | 0.1749 | 0.3729 | 0.469 | 0.639 | 4023.3 |
|  |  |  | zone | -2.5058 | 0.5744 | -4.362 | <0.001 |  |
| 4 | NBMM | date | intercept | 0.4320 | 0.1300 | 3.322 | 0.000894 | 4108.7 |
|  |  |  | zone | -2.3646 | 0.2151 | -10.991 | <0.001 |  |
| 5 | NBMM | date:cluster, cluster | intercept | -0.1579 | 0.3356 | -0.470 | 0.638 | 3935.8 |
|  |  |  | zone | -2.4618 | 0.5262 | -4.679 | <0.001 |  |
| 6 | NBMM | season:(date:cluster), date:cluster, cluster | intercept | -0.1579 | 0.3355 | -0.471 | 0.638 | 3937.8 |
|  |  |  | zone | -2.4618 | 0.5261 | -4.680 | <0.001 |  |
| 7 | NBMM | season:date, date | intercept | 0.4320 | 0.1300 | 3.322 | 0.000893 | 4110.7 |
|  |  |  | zone | -2.3646 | 0.2151 | -10.992 | <0.001 |  |
| 8 | NBMM | season:cluster, cluster | intercept | 0.1279 | 0.3596 | 0.356 | 0.722 | 4011.3 |
|  |  |  | zone | -2.4831 | 0.5568 | -4.459 | <0.001 |  |
| 9 | NBMM | season | intercept | 0.7511 | 0.1256 | 5.981 | <0.001 | 4192.1 |
|  |  |  | zone | -2.3457 | 0.1309 | -17.914 | <0.001 |  |
| 10 | NBMM | season | intercept | -682.6304 | 220.5750 | -3.095 | 0.001970 | 4181.3 |
|  |  |  | zone | -2.4271 | 0.1331 | -18.235 | <0.001 |  |
|  |  |  | season | -0.3716 | 0.1088 | -3.416 | 0.000635 |  |
|  |  |  | year | 0.3386 | 0.1093 | 3.099 | 0.001942 |  |
| 11^d^ | NBMM | year:(date:cluster), date:cluster, cluster | intercept | -0.08174 | 0.34388 | -0.238 | 0.812 | 3938.7 |
|  |  |  | zone | -2.47435 | 0.52763 | -4.690 | <0.001 |  |
|  |  |  | season | -0.16377 | 0.15528 | -1.055 | 0.292 |  |
| 12 | Linear | - | intercept | -644.4508 | 378.2197 | -1.704 | 0.08860 | 8341.4 |
|  |  |  | zone | -2.0537 | 0.1903 | -10.792 | <0.001 |  |
|  |  |  | season | -0.6016 | 0.1851 | -3.250 | 0.00118 |  |
|  |  |  | year | 0.3205 | 0.1874 | 1.710 | 0.08740 |  |
| 13 | Linear | - | intercept | 2.4447 | 0.1409 | 17.345 | <0.001 | 8342.4 |
|  |  |  | zone | -2.0070 | 0.1884 | -10.650 | <0.001 |  |
|  |  |  | season | -0.5695 | 0.1843 | -3.091 | 0.00203 |  |
| 14 | Linear | - | intercept | -519.8776 | 377.4364 | -1.377 | 0.169 | 8350.0 |
|  |  |  | zone | -2.0197 | 0.1906 | -10.596 | <0.001 |  |
|  |  |  | year | 0.2586 | 0.1870 | 1.383 | 0.167 |  |
| 15 | Linear | - | intercept | 2.1928 | 0.1153 | 19.02 | <0.001 | 8349.9 |
|  |  |  | zone | -1.9831 | 0.1888 | -10.50 | <0.001 |  |
| 16 | ZINB^e^ | - | intercept | -0.1627 | 0.0839 | -1.940 | 0.0524 |  |
|  |  |  | zone | 1.9723 | 0.1638 | 12.040 | <0.001 |  |
|  |  |  | season | 0.1039 | 0.1213 | 0.856 | 0.3920 |  |

^a^ S.E., standard error

^b^ AIC, akaike information criterion

^c^ NBMM, negative binomial mixed model

^d^ Best model selected with the 3^rd^ lowest AIC and variables of interest

^e^ ZINB, zero-inflated negative binomial
